# Proceedings of the Tennessee Dental Association, Seventh Annual Meeting, at Nashville, October 22nd and 23d, 1873

**Published:** 1873-12

**Authors:** L. G. Noel


### Proceedings of Societies.

### PROCEEDINGS OF THE TENNESSEE DENTAL ASSOCIATION, SEVENTH ANNUAL MEETING, AT NASHVILLE, OCTOBER 22d AND 23d, 1873.

### FIRST DAY, MORNING SESSION.

The Association met at the dental depot of Herman & Son, on Summer St., and was called to order at 10 o'clock by the President, Dr. J. C. Ross.

After prayer by Dr. L. C. Chisholm, the roll was called, and the following members answered to their names: J. C. Ross, S. J. Cobb, R. Russell, R. R. Freeman, W. H. Morgan, E. A. Herman and L. G. Noel, of Nashville; L. C. Chisholm, Franklin College; E. S. Chisholm, Tuscaloosa, Ala.; and Thomas Walsh, of Murfreesboro.

There were present later in the meeting, Drs. J. H. Webber, of Springfield ; Alex. Hartman, of Murfreesboro ; G. C. Sandusky, of Shelbyville ; T. B. Legare', of Clarksville ; J. H. Lassater, of Tullahoma ; T. J. Holden, of Gallatin ; Thomas Westerfield and J. S. King, of Nashville.

Reading of minutes of last meeting was dispensed with, and hours for meeting and adjourning fixed as follows : morning session, from 9 1-2 to 1 o'clock ; afternoon session, from 2 1-2 to 5 o'clock.

The name of Dr. J. S. King, of Nashville, was proposed for membership, and a ballot upon the same resulted in his unanimous election. Dr. T. J. Holden, of Gallatin, was also elected a member of the Society.

The Committce on Arrangements and Transportation now made their reports.

The report of the Committee on Dental Chemistry was called for. The Sec'y said that he and Dr. Chisholm were the only members of that Committee present, and in his own behalf he would say that the subject had been assigned him too late in the year for him to make the investigation he had designed to make ; and therefore he had not prepared a paper upon the subject. Had examined the reports of committees on this subject for many years past, and had been reluctantly brought to the conclusion that very little advancement was being made in this direction. Several theories had been put forth relative to the causes at work in the production of decay, but no positive light had been thrown upon the subject. These old questions had been recently unearthed, and discussed in dental societies ; and the advocates for the alkaline, acid and parasitic hypotheses had all had their say. We need more investigation, and less talk. He spoke of the chemical abrasion met with in the mouths of rheumatic patients, as affording an interesting field for experimentation and study. Thought that Dr. R. B. Todd's opinion, that rheumatism is a disease of the blood in which there is present some abnormal chemical agent in that fluid, derived some support from these cases.

Dr. L. C. Chisholm gave it as his opinion that carbonic acid was the destructive agent at work upon the teeth. He spoke of the work it performs in nature, in the disintegration of rocks and fertilization of soils; but referred especially to its readiness to combine with lime salts, and thought it capable of displacing the phosphoric acid of the lime phosphate of the teeth. Had not time to investigate the subject this year by experimentation, as he had hoped to do, and hence had not come prepared with a paper upon the subject. He spoke of the work which vegetables perform in the preparation of mineral substances for the building up of the animal tissues. Thought that mineral matter must be thus organized before it could be assimilated by animal tissues.

Dr. Morgan: "I know that this view has been advanced by modern physiologists; but I am not yet prepared to admit its truth. If it is true, why administer iron as a tonic? and how can that instinct which prompts animals to seek salt-springs be accounted for? This longing for the chloride of sodium is an instinct common to all animals, and they seek it wherever they can find it. Only a few plants supply it, and they grow upon the sea-coast; therefore they must get it from the inorganic world."

Dr. E. S. Chisholm: "I am a rheumatic myself, and my own mouth presents an example of such abrasion."

Dr. Freeman had a case under treatment, in which the affection could be traced to no such cause, and in which only the grinding surfaces of the two first inferior molars were involved. The patient was a young lady.

Dr. E. S. Chisholm thought the wear in many cases due to the eructation of hydrochloric acid from the stomach. Had met with it frequently in the mouths of dyspeptics.

Dr. Morgan has found these abraded teeth soft, though they may be of good color and look very hard.

Dr. Westerfield: "Is not this wear due to mechanical instead of chemical means? else why are not other surfaces attacked besides those exposed to trituration?"

Dr. King: "This view is in the main correct. It is true that the teeth are cupped out until their grinding surfaces will not come in contact in the acclusion of the jaws; but it is not nessary to preserve actual contact in order to keep up the wear—the friction of the food against these surfaces in mastication will do that. Chemical action never produces such polished surfaces as these teeth present. Such a surface cannot be produced by the action of an acid upon any substance."

Dr. Freeman called attention to the manner in which marble is polished by very dilute hydrochloric acid upon a flannel rag.

Dr. Russell said that chemical abrasion frequently commenced in eroded spots upon the surface.

This discussion was carried on, in a conversational way, up to time for adjournment. Adjourned to meet at 2 1-2 o'clock.

AFTENOON SESSION.

The Society met again at 2 1-2 o'clock, minutes of morning session were read and approved. The application of J. H. Lassater, of Tullahoma, for membership, was referred to the Executive Committee. The regular order of business was suspended to hear a paper on the "Treatment of Exposed Pulps," by Dr. J. S. King. After the reading of the paper, he presented a patient for examination, in whose mouth a number of teeth had been filled, and a number remained to be treated.

The case presented slight irregularity of the superior incisors ; the left inferior second bicuspid had been extracted, and the pulps in three first molars had been devitalized. Dr. King said he wanted an expression from those present as to the best mode of regulating the front teeth, and as to the advisability of extracting the sixth year molars. These had been filled with amalgam, were largely decayed and were crumbling about the edges of the fillings. The patient was *aet.* 17. This case was examined by all present and freely discussed.

Discussion of Dr. King's paper :

Dr. Morgan called for the reading of that portion of the Constitution concerning students, after which he said he only wanted to show that the paper was in accordance with the Constitution on that point.

Thought Dr. K. too radical when he advised the entire abandonment of all articles for the destruction of the pulp. Dr. L. C. Chisholm has had aching follow the application of oxy-chloride of zinc in nearly every instance, until he tried

Dr. King's method ; but since he first used it had perfect success.

Dr. Russell has found Dr. K's. method entirely satisfactory.

Dr. E. C. Chisholm also spoke in high terms of Dr. King's method of treating exposed pulps.

(The treatment alluded to here, as stated in the paper, is briefly as follows : The cavity is prepared as for a permanent filling of gold at once. A small quantity of white oxide of zinc is mixed with wood creosote and applied to the pulp, the cavity having been previously flooded with creosote. Just enough of the mixture is used to protect the pulp from the escharotic chloride, and after its application, the cavity is filled with the oxy-chloride. The operation may be completed and permanent filling of gold be inserted at this sitting, or deferred, as the operator thinks best.)

Dr. Morgan : "I have been using oxy-chloride of zinc for capping exposed nerves for six years. Dr. King's is only a modification of the method I have employed. I always used creosote or carbolic acid, flooding the cavity before filling with the oxy-chloride of zinc. Have capped nerves successfully in this way where suppuration and partial sloughing had occurred. There are cases, however, in which I am obliged to destroy the pulp, or resort to the forceps. If there are those who have acquired such a degree of skill in the treatment of these cases as never to fail of saving the nerve alive, I am willing to accord them all praise for their superior ability. I cannot do it in every case."

Dr. L. C. Chisholm asked for Dr. King's method of treating alveolar abscess. He replied that he used no disinfectant save creosote. Fills roots with Hill's stopping. In capping pulps, where the oxy-chloride would so fill up the cavity as to render the anchorage for the gold insufficient, he plants a little bit of the gold in it, while it is yet soft, having curved ends, and to this anchors the filling.

Dr. E. S. Chisholm had used oil of cloves and creosote, equal parts ; mixing the white oxide with this, he applied to the nerve as Dr. King uses his preparation. Had been equally successful with this.

Dr. Russell has used the aromatic sulphuric acid in the treatment of alveolar abscess, with encouraging results.

Dr. King was appointed to hold a clinic at his office to-morrow morning at 8 o'clock.

The Society now adjourned.

SECOND DAY, MORNING SESSION.

After the clinic at Dr. King's office, the Association came together again at 9 o'clock.

The discussion of Dr. King's paper was resumed.

Dr. Morgan: "I would ask if it is fair to deduce from Dr. King's paper the fact that he would do away with the use of the forceps entirely?"

Dr. King: "No: That sentiment is applied only to cases in which the pulp may be restored to health. I would extract dead ulcerating roots, where nature is evidently trying to get rid of them as foreign bodies."

The application of Dr. T. B. Legare', of Clarksville, for membership, was presented. The Executive Committee gave a favorable report of him, and upon a ballot being taken he was unanimously elected to membership.

The Committee on Operative Dentistry was called for, and, in default of a report, Dr. E. S. Chisholm read a paper on "Diagnosis."

A vote of thanks was accorded Prof. J. H. McQuillen, of Philadelphia, for the donation to the Society of a plaster model of a hypertrophied tooth.

The Committee on Publication now made their report. They stated that 2,000 copies of the pamphlet, "Care of the Teeth," had been published, and presented copies to the Society for examination.

A motion for the reading of the minutes of the last annual meeting was now put to the house, and carried. The minutes were lengthy and much time was consumed in reading them.

After this came the election of officers for the ensuing year, resulting in the following choice:

President, R. Russell, Nashville; 1st Vice-President, E. S. Chisholm, Tuscaloosa, Ala.; 2d Vice-President, J. S. King, Nashville; Rec. Sec'y., A. Hartman, Murfreesboro; Cor.

Sec'y., L. G. Noel, Nashville; Treasurer, R. R. Freeman, Nashville.

Executive Committee, L. C. Chisholm, Thos. Walsh and G. C. Sandusky.

Nashville was selected as the place for next meeting. Adjourned to meet at 2 1-2 o'clock.

AFTERNOON SESSION.

The Society met according to adjournment. Dr. J. H. Lassater's application was balloted upon, resulting in his election to membership. The newly elected President was now conducted to the chair, and upon taking his seat made a few remarks relevant to the occasion. The retiring President made a short address, in which he took a periscopic glance at the profession—its progress, present status and prospects for the future.

Dr. Morgan exhibited an apparatus he had used for regulating the superior incisor teeth, after which he read a paper on "The Diseases of the Antrum."

Discussion—Dr. Cobb: "I had myself an affection of the antrum, caused by the 2d left superior molar. The tooth was diseased two years, during which time there was discharge from the nose. There is a very curious fact connected with this subject, at least so far as my observation goes, and that is that nearly all these cases occur on the left side of the face. I saw a case in which a dentist, in his efforts to remove the roots of a molar tooth, had pushed them into the antrum—the floor of which was necrosed and nearly gone. The roots were afterwards discharged through the nose. I treated the case with a mixture of carbolic acid, glycerine and tinct. iodine. The patient recovered, but the opening into the antrum healed without closure. I had another case in which there was an opening upon the exterior of the face communicating with the cavity of the antrum. This case was relieved by the extraction of a tooth."

Dr. Morgan had noticed that the left side of the face was most frequently affected with diseased antrum.

Others concurred in this observation.

Dr. L. C. Chisholm detailed a case of fibrous tumor of the antrum which came under his observation. The tumor was removed by Prof. Gross of Louisville, and weighed eleven ounces

Dr. King detailed a case in which the trouble was caused by the death of the pulp in a second superior molar. Abscess had formed and pointed near the roots of the wisdom tooth. The patient's physician extracted the wisdom tooth, but this failed to give relief. It was at this stage he saw the case. All the symptoms of diseased antrum were present. The discharge from the nose, tenderness of the parts upon pressure, pain and flow of tears from the eye. Had sharp words with the physician on account of difference of opinion about the case. Finally convinced him, extracted tooth, opened cavity of antrum, and after a little treatment the patient was dismissed, cured.

Dr. E. S. Chisholm now read a short history of a case of exfoliation of a portion of the inferior alveolar process.

Dr. S. P. Cutler, Chairman of the Committee on Microscopy, presented a paper on that subject, giving the history and microscopy of a tooth (an inferior molar) in which the points of the fangs had become connected by osseous deposit.

The report of the Committee on Mechanical Dentistry was called for. No paper on that subject.

Dr. E. C. Chisholm said he wished to retract some remarks he had made in this Society a year ago. He had just commenced to experiment with celluloid then and was pleased with it. So expressed himself. Since that time his plates had come back to him warped out of shape, and, in many instances, with the teeth ready to drop off.

Dr. Cobb said his experience with celluloid coincided exactly with that of Dr. Chisholm, and he regarded it as unfit for a base for artificial teeth. He said that rubber was the best thing that he had tried yet, and advised all present to connect themselves with the "Tennessee Dental Protective League," and help to carry on the war against Bacon, Adjourned to meet 7 1-2 o'clock at in the evening.

EVENING SESSION.

Met according to adjournment at Dr. Freeman's office.

The Committee on "Materials for Filling Teeth" was called upon for their report. No paper on that subject.

Discussion of the subject followed.

Dr. Morgan: "I think it unnecessary to discuss any other material than gold. I have almost ceased to use soft gold, and prefer it strictly cohesive, in the majority of cases. I prefer that prepared by Geo. J. Pack, and known as Eureka Filling, to anything I have yet seen. I have been using the Globe Foil, semi-cohesive and cohesive, and am much pleased with it. It is exceedingly tough and does not become harsh when annealed. I prefer Nos. ranging from 5 to 40."

Dr. Freeman: "I have tried the Globe Foil and like it very much. I prefer the semi-cohesive, and usually anneal the strips slightly before attempting to weld. Of Pack's preparations, I prefer the Crystal Pellets."

Dr. E. S. Chisholm: "I always start my fillings with soft foil. I prefer this to cutting retaining pits for the first pieces of gold. After securing a foundation in this way, I build up with cohesive gold; using serrated points until the filling is nearly completed, then changing them for smoother ones."

Dr. Cobb: "Dr. L. C. Chisholm says he is on the old fogy line—guess he wants company, and I'll go with him. I use soft gold now more than I ever did before. I used to build up and restore contour, doing all those fancy operations, but have pretty much abandoned that practice of late years. I think Abbey's Foil superior to anything I have seen. I have seen teeth preserved for years with soft foil. When we get at the truth we can go no further. If gold could be made as soft as lead and so as to stand the wear, it would be better. Cohesive gold lacks the lateral expansion which can be secured with the non-cohesive. I like the non-cohesive Globe Foil; it is certainly the toughest gold I have seen. I don't use the rubber dam much, only occasionally. The reason I am not making contour operations now is, that they will come back on me to be done over. I think amalgam will sometimes preserve a tooth. It has been said here that we need hardly discuss any other material than gold, but I have seen teeth preserved for years with amalgam. It has a place and ought to be used sometimes."

Dr. Ross: "I like Ney's Foil, Nos. 5 and 6. Have abandoned the heavy foils. I use the mallet and rubber dam constantly."

Dr. King: "I prefer No. 4, and never use heavier than 6. As regards other materials than gold for filling teeth, if I had my choice I would abandon them all. About 7-8 of all the amalgam fillings made will fail in from 2 1-2 to 3 years."

Dr. Freeman: "Dr. Elisha Wright made contour fillings of soft gold 35 years ago. He always passed it through a flame before using, however. I am inclined to think that these old contour fillings that are being fished up from 35 or 40 years ago would turn out that way if inquiry was made. They were made of cohesive gold after all."

Dr. Russell: "You can put more gold into a tooth with a mallet than ought to go in, and where fillings are made so dense the enamel will scale off. The tooth will not stand the expansion of so dense a mass when heat is appled."

Dr. L. C. Chisholm: "Dr. Russell's statement is correct. Too much gold can be put into a tooth when condensed with mallet force. More gold can be put into a cavity in this way than by melting and casting. The enamel will scale off. I have seen this crumbling, where I was sure it was the result of the expansion of the metal."

Dr. King: "The idea Dr. Chisholm has advanced is a new one to me, and I am too incredulous to accept it as true until I have seen it demonstrated. I think if he will put it to actual test he will find he is in error."

Dr. Morgan: "The great trouble with most men in the use of cohesive gold lies in the fact that they ignore the rubber dam. They don't know how to use it."

Dr. Legare': "I agree with Dr. Morgan in regard to the use of the rubber. I apprehend that it makes little difference whose preparation of gold you use, provided you keep it dry."

The report of the Committee on Publication was again referred to, and the price of the pamphlet, "Care of the Teeth,"

fixed at $5 per 100 copies—$6 with the card of the dentist printed on the back.

A committee was appointed to get up another pamphlet of like character, to be presented to the Society in manuscript at its next meeting. The committee consists of Drs. S. J. Cobb, A. Hartman, J. C. Ross, R. Russell, A. F. Claywell, L. C. Chisholm, S. P. Cutler, J. S. King and E. S. Chisholm.

The time of meeting was again changed and the Society will hereafter meet in annual session on the 4th Wednesday in June.

A vote of thanks was accorded Drs. Herman and Freeman for furnishing place of meeting. A vote of thanks was also accorded the Secretary for the manner in which he had kept the minutes.

The Society now adjourned to meet in this city on the 4th Wednesday in June, 1874. L. G. NOEL, *Rec. Sec'y.*

